# Detection and characterization of meat adulteration in various types of meat products by using a high-efficiency multiplex polymerase chain reaction technique

**DOI:** 10.3389/fnut.2022.979977

**Published:** 2022-09-16

**Authors:** Caijiao Yang, Guowei Zhong, Song Zhou, Yingqi Guo, Daodong Pan, Sha Wang, Qianqian Liu, Qiang Xia, Zhendong Cai

**Affiliations:** ^1^Key Laboratory of Animal Protein Deep Processing Technology of Zhejiang Province, College of Food and Pharmaceutical Sciences, Ningbo University, Ningbo, China; ^2^Center for Global Health, School of Public Health, Nanjing Medical University, Nanjing, China; ^3^Key Laboratory of Vector Biology and Pathogen Control of Zhejiang Province, Huzhou University, Huzhou, China; ^4^Key Lab of Clean Energy and Green Circulation, College of Chemistry and Material Science, Huaibei Normal University, Huaibei, China

**Keywords:** multiplex PCR, meat species, molecular authenticity, primer specificity, commercial foodstuffs

## Abstract

Identification of meat authenticity is a matter of increasing concerns due to religious, economical, legal, and public health reasons. However, little is known about the inspection of eight meat species in one tube reaction due to technological challenge of multiplex polymerase chain reaction (PCR) techniques. Here, a developed multiplex PCR method can simultaneously authenticate eight meat species including ostrich (753 bp), cat (564 bp), goose (391 bp), duck (347 bp), chicken (268 bp), horse (227 bp), dog (190 bp), and sheep (131 bp). The detectable deoxyribonucleic acid (DNA) contents for each target species was as low as 0.01 ng in both raw and heat-treated meat or target meat down to 0.1% (w/w) of total meat weight reflecting high stability of the assay in heat processing condition, indicating that this method is adequate for tracing meat origin in real-world meat products, which has been further validated by authenticity assays of commercial meat products. Overall, this method is a powerful tool for accurate evaluation of meat origin with a good application foreground.

## Introduction

Some animal species such as ruminant (beef and sheep) and poultry (chicken and duck) are the popular meat resource along with the top consumption rate at all corners of the world, which can supply essential nutrients especially for the richest protein source (1, 2). However, the growing demand for animal protein has further exacerbated the incidence of meat frauds in animal protein-based foodstuffs, which has caused severe global issue (3–6). Meat adulteration, whether deliberately or unintentionally, not only breaks market rules but also violates ethical norms and religious laws. For example, pork consumption is strictly restricted in Islam and Judaism; beef is prohibited for the Hindus (7, 8). Most importantly, meat frauds risk the food safety and even threaten public health such as metabolic disorders, allergies and infectious diseases, because both inedible and edible meat products can occasionally elicit allergic reactions especially for sensitized patients (5, 6, 9–11). Based on this, meat molecular detection is important to protect consumers from being deceived, and ensure food safety in dietary practices.

Many analytical methods have been developed for meat authentication. Traditionally, protein-based methods such as enzyme-linked immunosorbent assay (ELISA) are more reliable to identify animal species, which have gained huge popularity in food business based on the simplicity and low cost (12). However, denaturation of protein under extreme thermal treatment prevents the accuracy of meat detection during food processing and let such techniques unsuitable for meat authentication especially in processed meat products (13, 14). Unlike proteins and peptides, deoxyribonucleic acid (DNA) molecules possess more stability against harsh thermal and chemical treatments (15, 16). Thus, DNA-based methods are more favorable for meat species detection in various meat samples. The polymerase chain reaction (PCR) approaches are well known DNA-based methods for detecting animal origin in foodstuffs, which include species-specific PCR, multiplex PCR, PCR-RFLP, PCR-RAPD, DNA barcoding, real-time PCR, and multiplex PCR (3). Among them, multiplex PCR assays are cost-effective and time-saving for simultaneous identification of multiple meat species. In particular, mitochondrial DNA (mtDNA) has multiple copies showing intraspecies conservation and interspecies polymorphism, which are proper target sites for species-specific primers. Moreover, DNA stability increases the chance of survival in processed products even at the condition of heat processing treatments, indicating that target sequences of mtDNA designated as target primers are adequate for meat inspection in heat processing foodstuffs. As reported, mtDNA sequences such as cytochrome b, 12S and 16S rRNA, D-loop, ATPase subunits 6 and 8, NADH dehydrogenases are common targets for designing species-specific primers of multiplex PCR (3, 17–21).

In this study, using mtDNA genes including 16S rRNA, NADH dehydrogenase subunit 1, 3, and 5, Cytochrome c oxidase subunit I, III, ATPase subunits 6, and D-loop as targets, species-specific primers for eight animal species ostrich, cat, goose, duck, chicken, horse, dog, and sheep were designed and then checked through the analyses of cross-reactivity, specificity, sensitivity, and robustness. Using the optimized PCR system, an octuplex PCR assay was ultimately developed with eight sets of species-specific primer pairs, which can inspect eight meat origin in both raw and processed meat products. To our knowledge, little is known about the molecular authentication of eight animal ingredients in one PCR reaction due to technological challenge of this multiplex PCR technique. Moreover, this method has been validated to be adequate for assessment of meat fraud incidences in commercial foodstuffs.

## Materials and methods

### Samples collection and deoxyribonucleic acid extraction

Fresh pure meat of 17 target species were purchased from local market, farm as well as online supermarket platform, which included 14 land animals of sheep (*Ovis aries*), dog (*Canis lupus*), horse (*Equus caballus*), chicken (*Gallus gallus*), duck (*Anas platyrhynchos*), turkey (*Meleagris gallopavo*), pigeon (*Columba livia*), camel (*Camelus bactrianus*), rabbit (*Oryctolagus cuniculus*), ostrich (*Struthio camelus*), cattle (*Bos taurus*), cat (*Felis catus*), goose (*Anser cygnoides*), and pig (*Sus scrofa*) as well as 3 aquatic species of small yellow croaker (*Larimichthys polyactis*), tuna (*Thunnus orientalis*) and black carp (*Mylopharyngodon piceus*). In addition, 30 commercial samples including raw and heat processing of meat balls (5), meat slices (5), kebab (3), sausages (6), jerky (5), and cutlets (4) were purchased from markets or online supermarket platform. All samples were transported under ice-chilled condition and stored at –80^°^C until further use for DNA extraction. Genomic DNA was isolated from each sample using a Beyotime kit (D0063, Beyotime Biotechnology Co., Ltd., Shanghai, China). The purity and concentration of extracted DNA were measured by a NanoDrop 2000 spectrophotometer (NanoDrop 2000, UV–Vis spectrophotometer, United States) (22).

### Design of species-specific primers

The primers for each species were designed by targeting at mitochondria sequences including D-loop of sheep (GenBank Accession No. KP702285.1), ATPase subunits 6 of dog (MN181404.1), cytochrome c oxidase subunit I of horse (MN187574.1), NADH dehydrogenase subunit 3 of chicken (MK163565.1), NADH dehydrogenase subunit 1 of duck (MK770342.1), cytochrome c oxidase subunit III of goose (KJ124555.1), NADH dehydrogenase subunit 5 of cat (MT499915.1) and 16S rRNA of ostrich (Y12025.1). Combined the analyses of Oligo 7.0 and BLAST programs, species-specific primers were designed and optimized according to physical parameters of cross-reactivity, melting temperature, self-complementarity as well as secondary structures. The specificity of species-specific primers was confirmed by alignment against animal species including 14 land animals and 3 aquatic species as aforementioned by a ClustalW sequence alignment program and the MEGA6 software. Finally, the cross reaction was determined by species-specific primer pair against a total of 16 non-target animal species through simplex PCR assays. The information of primer sets in detail was shown in Table 1. The designed primers were synthesized by Shanghai Sangon Biological Engineering Technology and Services Co., Ltd. (Shanghai, China) (22).

**TABLE 1:** Oligonucleotide primers for meat species used in this study.

Primers	Genes	Sequence (5′–3′ direction)	Amplicons (bp)	References or source
Ostrich	16S rRNA	TAACTTACCCCTCCCGGCATC	753	This study
		AAACGAGGATCAGTTGGTTGCAG		
Cat	NADH dehydrogenase subunit 5	AAACCAATGCCCTTCACCACT	564	This study
		TATCGATGCGGACTTTTGGCTC		
Goose	Cytochrome c oxidase subunit III	CAAGGCCATCACACTCCCACA	391	This study
		AGAAGGTAGATCCGTAGACGCTA		
Duck	NADH dehydrogenase subunit 1	CCCGTTCTCACTAGTAGACCT	347	This study
		GTTCAGACTCGCCCTCCGTTA		
Chicken	NADH dehydrogenase subunit 3	ATCCTAAACTTTCTTCTCGCTCA	268	This study
		TCCCAGTGTAAGGAGGCTAA		
Horse	Cytochrome c oxidase subunit I	ATTGGAGCACCTGATATAGCTT	227	This study
		ATGGCACCTAAAATCGAGGACA		
Dog	ATPase subunits 6	CCAAGGCACCCCTCTTCCC	190	This study
		AAAAGTGATAAAAGCTGTGGTCG		
Sheep	D-loop	ATACAACACGGACTTCCCACT	131	This study
		CTCGCTTAGCACATTCAAGACAG		
Eukaryotes	12S rRNA	CAACTGGGATTAGATACCCCACTAT	456	(33)
		GAGGGTGACGGGCGGTGTGT		
Eukaryotes	16S rRNA	AAGACGAGAAGACCCTATGGA	240	(27)
		GATTGCGCTGTTATCCCTAGGGTA		
Eukaryotes	18S rRNA	AGGATCCATTGGAGGGCAAGT	99	(34)
		TCCAACTACGAGCTTTTTAACTGCA		

### Simplex and multiplex polymerase chain reaction assays

A simplex PCR assay was constructed for target species with individual set of species-specific primers. PCR reaction of a final 25 μL volume contains 2.5 μL of 10 × EasyTaq^®^ Buffer, 2 μL of 2.5 mM dNTPs, 0.5 μL of 5 U/μL EasyTaq DNA Polymerase, 0.5 μL of 10 μM each primer, genomic DNA and ddH_2_O. PCR amplification with deionized water in place of template DNA as a negative control to check any DNA contamination in each reaction system. PCR reaction was conducted by the initial denaturation at 94^°^C for 5 min, followed by 34 cycles of denaturation at 94^°^C for 30 s, annealing at 63^°^C for 30 s and extension at 72^°^C for 45 s, and a final extension at 72^°^C for 5 min in T100™ Thermal Cycler (Bio-Rad, Germany). For multiplex PCR assays, they were started from duplex to octuplex PCRs with optimized PCR system. PCR reaction system (25 μL) included 2.5 μL of 10 × EasyTaq^®^ Buffer, 2 μL of 2.5 mM dNTPs, 0.5 μL of 5 U/μL EasyTaq DNA Polymerase, 0.5 μL of 10 μM each primer pair of eight species, DNA mixture of eight species at the indicated concentration and refilled ddH_2_O to 25 μL. PCR amplification was performed by T100™ Thermal Cycler under the same condition of PCR amplification as simplex PCR. PCR products were electrophoresed on a 5% agarose gel by using 4S GelRed Nucleic Acid Stain, and visualized by Gel DocTM XR + System with Image LabTM Software (BIO-RAD) (23).

### Tests for specificity, sensitivity, and reproducibility

The specificity of each primer pair was individually checked by PCR assays against individual sample of all species (camel, pigeon, chicken, duck, horse, cattle, pork, turkey, goose, sheep, rabbit, ostrich, dog, cat, small yellow croaker, tuna, and black carp). Simplex and multiplex PCR results were run on 5% agarose gel and then visualized for the proper amplification. To determine the sensitivity of the multiplex assay, a series of PCR amplification were performed by using serial dilutions of the premixed genomic DNA templates of eight target species in one reaction. Ten concentrations of all target templates ranging from 10 to 0.01 ng were used for detecting the limit of detection (LOD). The amplified products were run on a 5% agarose gel for separation and visualization. To check the reproducibility of species-specific primers, raw meat samples for each species were deliberately subjected to heat processing treatment of boiled (97–99^°^C, 30 min) and microwave-cooked (750 W, 10 min) patterns. The robustness of PCR assay was evaluated with genomic DNA extracted from heat processing samples (22).

## Results

### Specificity of polymerase chain reaction assay

For all applied PCR systems, specificity is a prerequisite for multiplex assays. To construct a multiplex PCR assay, species-specific primers were designed through Oligo 7.0 and BLAST programs. As expected, PCR amplification with species-specific primers produced target bands with the predicted size of 753, 564, 391, 347, 268, 227, 190, and 131 bp for ostrich, cat, goose, duck, chicken, horse, dog, and sheep, respectively (Supplementary Figure 1A). As positive controls, three universal eukaryotic primer pairs targeting 18S, 16S, and 12S rRNA produced the predicted size of 99, 240, and 456 bp with similar intensities in all reaction tubes (Supplementary Figure 1B), suggesting the presence of high-quality DNA in all meat samples. As expected, the target bands were successfully amplified with target DNA isolated from a single species in the presence of premixed primers of all eight species but not seven non-target species (Supplementary Figure 1C), suggesting that primers designed for each species can specifically amplify target species. This conclusion was further supported by the assay that the target bands were generated by each set of species-specific primers in the presence of DNA mixture of all eight but not seven non-target species (Supplementary Figure 1D). In addition, cross-reaction of PCR amplification with each primer pair was further examined, which showed no cross-reactivity against sixteen non-target species indicated (data not shown). Collectively, the results demonstrated that the designed species-specific primers are adequate for food inspection.

### Sensitivity of multiplex polymerase chain reaction assay

**FIGURE 1:**
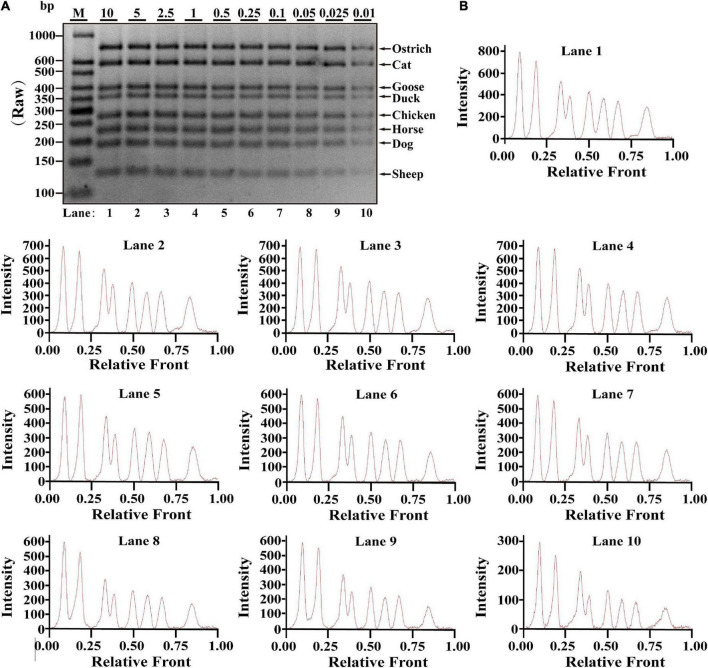
Validation of the sensitivity of multiplex PCR assay in raw meat samples. **(A)** Gel image of PCR fragments amplified by multiplex PCR using premixed DNA templates of eight species (10, 5, 2.5, 1, 0.5, 0.25, 0.1, 0.05, 0.025, and 0.01 ng) with species-specific primers of eight meat species in a single PCR reaction. **(B)** The corresponding electropherogram of gel image represented ostrich, cat, goose, duck, chicken, horse, dog, and sheep in each lane. Lanes 1–10 are presented with labels (10, 5, 2.5, 1, 0.5, 0.25, 0.1, 0.05, 0.025, and 0.01) in **(A)**. The value of number at the horizontal line means the relative position of peaks distant from the top of agarose gel. The value of number at the vertical line means the fluorescent intensity of DNA-bound dyes using 4S GelRed Nucleic Acid Stain. Lane M is ladder DNA.

Based on simplex PCR system optimized, multiplex PCRs were gradually constructed and an octuplex PCR method was ultimately developed with eight pairs of species-specific primers. Serial dilution of each meat DNA ranging from 10 to 0.01 ng was used to determine the sensitivity of this assay in one PCR reaction. As shown in Figure 1A, PCR products of ostrich, cat, goose, duck, chicken, horse, dog, and sheep were availably detected from 10 to 0.01 ng DNA. Electropherograms were drawn from the bands by using Image Lab™ Software, in which the intensities of peak patterns reflected the brightness of bands. As shown in Figure 1B, intact peaks patterns for ostrich, cat, goose, duck, chicken, horse, dog, and sheep with gradually decreased intensities of peaks were observed from lane 1 to 10, suggesting that available inspection for all target species can be achieved at the low level of 0.01 ng DNA. Therefore, LOD of this octuplex PCR method was presumably 0.01 ng DNA.

To determine the availability of this method in real-word foodstuffs, model sheep adulteration was constructed by simultaneous addition of seven meat tissues including ostrich, cat, goose, duck, chicken, horse and dog at 0.1, 0.25, 0.5, 1, 2.5, 5, or 10% of total weight, respectively. Next, the samples were subjected to genomic DNA extraction for multiplex PCR amplification. the specific amplicons for each species were clearly displayed even at target meat percentage of 0.1% (Supplementary Figures 2A,B).

### Validation of reproducible multiplex assay under heat-treated meat materials

**FIGURE 2:**
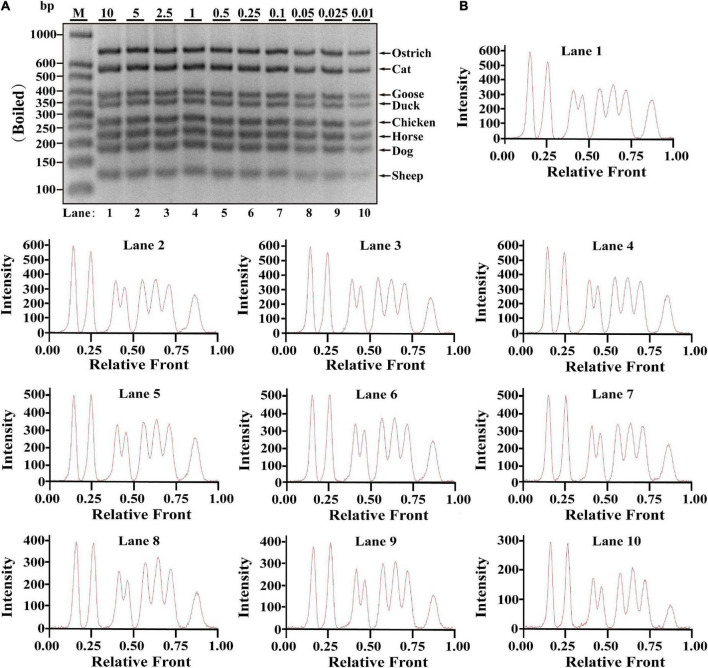
Validation of the sensitivity of multiplex PCR assay in boiling meat samples. **(A)** Gel image of PCR fragments amplified by multiplex PCR using premixed DNA templates of eight species (10, 5, 2.5, 1, 0.5, 0.25, 0.1, 0.05, 0.025, and 0.01 ng) with species-specific primers of eight meat species in a single PCR reaction. **(B)** The corresponding electropherogram of gel image represented ostrich, cat, goose, duck, chicken, horse, dog, and sheep in each lane. Lanes 1–10 are presented with labels (10, 5, 2.5, 1, 0.5, 0.25, 0.1, 0.05, 0.025, and 0.01) in **(A)**. The value of number at the horizontal line means the relative position of peaks distant from the top of agarose gel. The value of number at the vertical line means the fluorescent intensity of DNA-bound dyes using 4S GelRed Nucleic Acid Stain. Lane M is ladder DNA.

**FIGURE 3:**
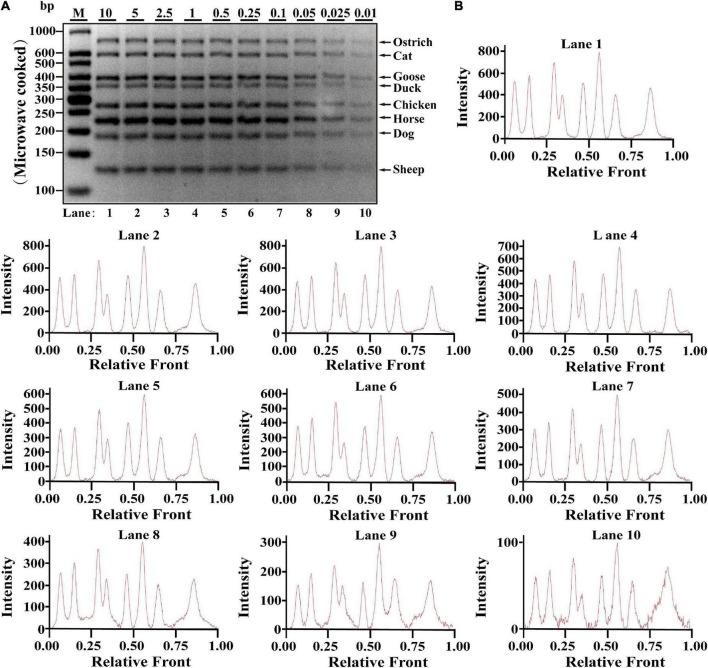
Validation of the sensitivity of multiplex PCR assay in microwave-cooking meat samples. **(A)** Gel image of PCR fragments amplified by multiplex PCR using premixed DNA templates of eight species (10, 5, 2.5, 1, 0.5, 0.25, 0.1, 0.05, 0.025, and 0.01 ng) with species-specific primers of eight meat species in a single PCR reaction. **(B)** The corresponding electropherogram of gel image represented ostrich, cat, goose, duck, chicken, horse, dog, and sheep in each lane. Lanes 1–10 are presented with labels (10, 5, 2.5, 1, 0.5, 0.25, 0.1, 0.05, 0.025, and 0.01) in **(A)**. The value of number at the horizontal line means the relative position of peaks distant from the top of agarose gel. The value of number at the vertical line means the fluorescent intensity of DNA-bound dyes using 4S GelRed Nucleic Acid Stain. Lane M is ladder DNA.

Since heat-processing treatments might cause DNA degradation, validation of PCR assay in terms of stability is essential for heat-treated samples prior to applying the technique on commercially processed food products, which was evaluated using DNA extracted from heat-processing samples of boiled and microwave-cooked treatments, respectively. For boiling meat samples, eight target bands were obviously observed at the range of 0.01–10 ng DNA; meanwhile, intact peak patterns for eight species were found in lanes 1–10 (Figures 2A,B), drawing a conclusion that LOD of this method was 0.01 ng DNA for boiling meat tissues. Using the same multiplex PCR system, template DNA from microwave-cooking meat tissues was used for assessing the availability of this multiplex PCR technique. Combined the analyses of agarose gel and electropherogram for microwave-cooking samples in Figures 3A,B, LOD of this method was about 0.01 ng DNA similar to that of boiling meat tissues. Taken together, the threshold value for inspection of heat processing meat was about 0.01 ng DNA.

### Application of multiplex polymerase chain reaction assay on commercial meat products

The applicability of this assay was checked with a total of 30 popular consumption products in food items of meat balls, meat slices, kebab, sausages, cutlets, and jerky. As shown in Supplementary Table 1, most samples contained the same meat origin as declared on the label. However, some products had contaminated with extra ingredients unlabeled. As illustrated, 3 of 10 (30%) sheep samples, 3 of 10 (30%) horse samples, and 2 of 10 (20%) ostrich samples had been validated to adulterate with meat ingredients unlisted. From the survey, some mislabeling samples often contaminated poultry meat such as duck, chicken, and goose, which were undeclared on the product labels. The mislabeling of meat products may be intentionally contaminated with cheaper meats for economic profit or unintentionally cross-contaminated in the production chain. For unintentional contamination, the sensitive method is necessary to monitor a little amount of meat contamination. From this survey, the proposed technique can be availably applied to monitor and control meat contamination.

## Discussion

Multiplex PCR techniques are highly favorable for the inspection of multiple targets in a single platform, which have gained huge popularity in food business based on the simplicity and low cost through simple agarose gel analysis (24–26). However, mutual interference of components such as templates and primers would become more complex with the increase of more primers and multiplicity of multiplex PCR reaction, which often cause lower efficiency and even the failure of amplification (8), indicating that multiplex PCRs are often subjected to technological challenge. Through the survey of multiplex PCRs recently published in Supplementary Table 2, multiplex PCRs generally detect less than eight animal origin in one-tube reaction platform. To our knowledge, there is only one report for monitoring eight meat ingredients through one-tube multiplex PCR method with the help of universal primers (27). Here, this proposed method can identify eight meat origin by eight sets of highly specific primers without extra universal primers in one tube reaction.

The specificity of primers is a prerequisite for multiplex PCR assays. To obtain a specific multiplex PCR assay, the primers should specifically match the target species, and have huge mismatches with non-targets (19, 28). Accordingly, the feature of primers is crucial for accurate authentication of meat species. By Oligo 7.0 and BLAST programs, target primers showed more stringent specificity and shared similar melting temperature to that of other targets ensuring to anneal with target templates under the same set of PCR conditions. As reported, even a single base pair that mismatches at the 3′ end of the primers with target DNA might interfere with the efficiency of PCR amplification (29). In this regard, target primers were stringently evaluated on base mismatches within primer annealing sites and then were aligned *in silico* against 16 other non-target species as mentioned. The sequences of each primer pair completely match with target species. Furthermore, the specificity of target primers was confirmed based on species-specific amplification of PCR assays. Notably, species-specific primers produced target bands with differential intensities under the same PCR condition, indicating that target primers have different amplification efficiency in this multiplex PCR system.

Referring to multiplex PCR assays (19, 24, 30), serial dilution of each meat DNA ranging from 10 to 0.01 ng was used to determine LOD of this method in one PCR reaction. Since it generated weaker bands for some species at the concentration of 0.01 ng DNA (Figures 1–3), lower DNA levels of each meat such as 1 pg or 0.01 pg were not further tested for LOD. Compared to LOD of multiplex PCR assays varying from 1 pg to 0.32 ng (Supplementary Table 1), LOD of this method is as low as 0.01 ng DNA in various meat samples of raw, boiled and microwave-cooked meat materials, suggesting that this method is qualified for monitoring meat resource. However, over-representation of certain species in an unknown sample might disguise the low presence of another one and generate a false-negative result. Most importantly, determination of LOD was accomplished by three independent experiments. In addition, economic benefits are a critical factor for the substitution of expensive and high-quality meat with inferior and low-cost ones and therefore the amounts of adulterated ingredients should be easily detected in real-word foodstuffs. The reproducibility assay reflected high stability of the method even for samples undergoing harsh heat-processing condition, which provides substantial evidences for the availability of the application of this PCR assay on commercial meat products (Figures 2, 3). Consistent with other reports (30–32), this multiplex PCR method demonstrates that meat fraud with cheap or poor-quality meat has become a commonplace in real-world foodstuffs (Supplementary Table 1), suggesting that this method is indeed adequate for identification of meat species in actual adulteration event due to its high sensitivity. Overall, the proposed octuplex PCR technique is a powerful tool for accurate evaluation of meat origin, which is crucial to safeguard consumers from meat fraud and contributes to establish discipline in food business.

## Conclusion

This study provides a reliable, low-cost, and rapid approach, which offers unambiguous detection and discrimination of eight animal species. Compared to multiplex PCRs documented, the detectable mtDNA contents for each target species were as low as 0.01 ng DNA in various meat materials so that the proposed multiplex PCR is highly promising for meat authentication in actual adulteration event, which is also easily implemented by simple agarose gel analysis without specific sophisticated equipment. The availability of the method has been corroborated by the application of multiplex PCR on commercial meat products. Therefore, molecular authentication or molecular traceability of meat origins through this multiplex PCR technique has provided an accurate evaluation of meat ingredients in real-world foodstuffs.

## Data availability statement

The raw data supporting the conclusions of this article will be made available by the authors, without undue reservation.

## Author contributions

SW, QL, and ZC: conception and design of the investigation and work. CY, GZ, SZ, YG, and ZC: completion of the experiments. CY, GZ, DP, QX, SW, QL, and ZC: evaluation and analysis of the results. CY, GZ, SW, QL, and ZC: manuscript writing. CY, GZ, SZ, YG, SW, QL, and ZC: final approval of manuscript. All authors contributed to the article and approved the submitted version.
