# Supplementary material for: Detection and characterization of meat adulteration in various types of meat products by using a high-efficiency multiplex polymerase chain reaction technique
Source: Front Nutr. 2022 Sep 16;9:979977. doi: 10.3389/fnut.2022.979977 (PMC9525214; doi:10.3389/fnut.2022.979977)
Supplement: Supplementary file 1 [file Presentation_1.pdf]

Detection and characterization of meat adulteration in various types of meat products by using a high-efficiency multiplex polymerase chain reaction technique

Caijiao Yang[1,#], Guowei Zhong[2,#], Song Zhou[1], Yingqi Guo[1], Daodong Pan[1], Sha Wang[3,*], Qianqian Liu[4,*], Qiang Xia[1], Zhendong Cai[1,*]

Supplementary Material

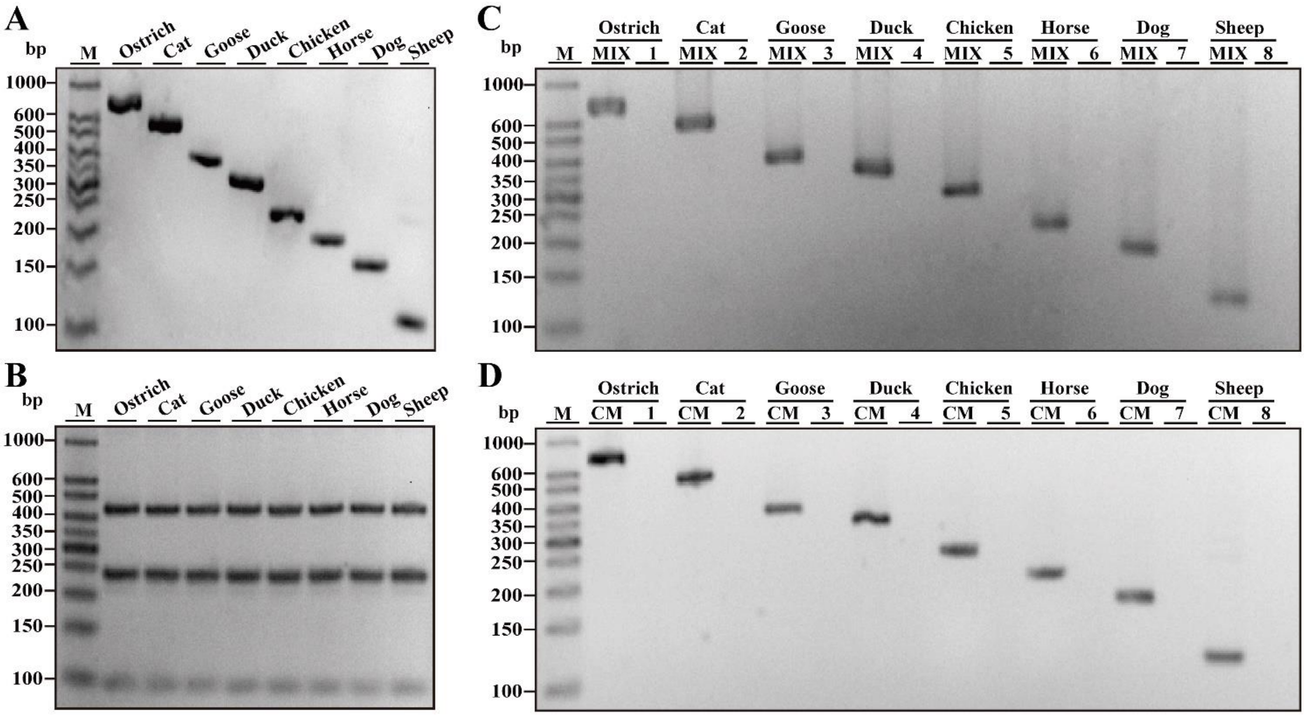

Supplementary Figure 1. Verification of primer specificity with conventional simplex PCR. (A) Simplex PCR detection using species-specific primers for ostrich, cat, goose, duck, chicken, horse, dog and sheep origin and respective genomic DNA as a template. (B) PCR amplification with premixed universal primers of eukaryotic 12S rRNA, 16S rRNA and 18S rRNA genes for each meat species, respectively. (C) PCR amplification using individual template DNA from ostrich, cat, goose, duck, chicken, horse, dog and sheep species. MIX, a mixture of eight primer pairs of ostrich, cat, goose, duck, chicken, horse, dog and sheep species; 1–8, a mixture of seven primer pairs of seven nontarget species. (D) PCR amplification with species-specific primers for ostrich, cat, goose, duck, chicken, horse, dog and sheep species. CM, a complete mixture of eight species including ostrich, cat, goose, duck, chicken, horse, dog and sheep; 1–8, a complete DNA mixture of seven meat species except target species. Lane M is ladder DNA.

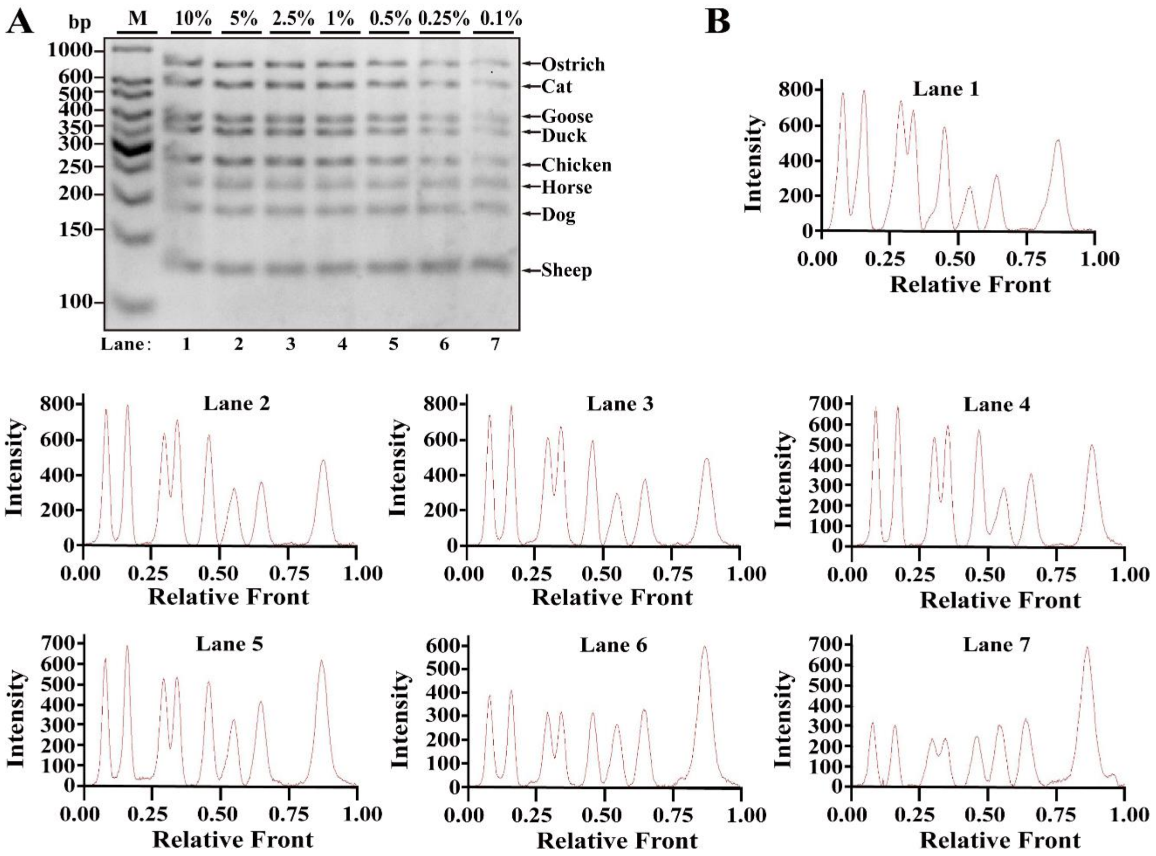

Supplementary Figure 2. Validation of the sensitivity of multiplex PCR assay in meat model mixtures. (A) Gel image of PCR fragments amplified by multiplex PCR using model mixtures of ostrich, cat, goose, duck, chicken, horse and dog added to sheep at 10%, 5%, 2.5%, 1%, 0.5%, 0.25% and 0.1% of total weight with species-specific primers of eight meat species in a single PCR reaction. (B) The corresponding electropherogram of gel image represented ostrich, cat, goose, duck, chicken, horse, dog and sheep in each lane. Lanes 1–7 are presented with labels (10%, 5%, 2.5%, 1%, 0.5%, 0.25% and 0.1%) in (A). The value of number at the horizontal line means the relative position of peaks distant from the top of agarose gel. The value of number at the vertical line means the fluorescent intensity of DNA-bound dyes using 4S GelRed Nucleic Acid Stain. Lane M is ladder DNA.

Supplementary Table 1. Results of PCR assay performed on commercial meat products

Products	Number	Labelled	Detected species								Adulteration
			Sheep	Dog	Horse	Chicken	Duck	Goose	Cat	Ostrich	
**Sheep**	10										3(30%)
meat balls	5	sheep	5/5			1/5[a]	1/5[a]	1/5[b]			
meat slices	2	sheep	2/2								
kebab	3	sheep	3/3			1/3[a]	1/3[a]				
**Horse**	10										3(30%)
meat slices	2	horse			2/2						
sausages	6	horse			5/5	1/5[a]	1/5[a],1/5[b]				
jerky	2	horse		1/3[a]	3/3						
**Ostrich**	10										2(20%)
cutlets	4	turkey						4/4	1/4[b]		
meat slices	3	turkey				1/4[a]		3/3			
jerky	3	turkey						3/3			

In each row, meat samples labeled with same letter (a or b) represent the identical meat samples, whereas different letters indicate a difference in meat samples

Supplementary Table 2. Comparative analysis of multiplex PCR assays for the identification of species origin of meat.

Multiplex PCR type	Species number	Detection items	Detection limit	Detection method	Reference or source
Multiplex	8	ostrich, cat, goose, duck, chicken, horse, dog, sheep	0.01 ng DNA or 0.1% for each species	Gel	This study
Octuplex	8	dog, chicken, cattle, pig, horse, donkey, fox, rabbit	0.05 ng/μL DNA	Gel	(1)
Septuple	7	turkey, goose, pig, sheep, beef, chicken, duck	0.01-0.05 ng DNA	Gel	(2)
Heptaplex - RFLP	7	beef, buffalo, chicken, duck, goat, sheep, pork	0.5% for each species	Chip	(3)
Multiplex	2	cattle, horse	0.05 ng DNA	Gel	(4)
Multiplex	3	chicken, duck, goose	0.05 ng DNA or 1% for each species	Gel	(5)
Multiplex	3	chicken, turkey, duck	1 pg for each species	Gel	(6)
Quadruple	4	beef, pork, mutton, duck	0.1 ng DNA	Gel	(7)
Multiplex	4	chicken, duck, pork, beef	0.05% for each species	Gel	(8)
Quadruple	4	fox, mink, or raccoon in beef, mutton	1% for each species	Gel	(9)
Multiplex	4	rat, fox, duck, sheep	0.05 ng/μL DNA	Gel	(10)
Pentaplex	5	dog, duck, buffalo, goat, sheep	0.1-0.32 ng DNA	Gel	(11)
Multiplex	5	cat, dog, pig, monkey, rat	0.01–0.02 ng DNA	chip	(12)
Multiplex	5	sheep/goat, bovine, chicken, duck, pig	0.5 ng DNA	Gel	(13)
Hexaplex	6	horse, soybean, sheep, poultry, pork, cow	0.01% for each species	Gel	(14)
Multiplex	6	goat, chicken, cattle, sheep, pig, horse	0.25 ng DNA	Gel	(15)
Multiplex	6	mutton, pork, duck, chicken, horse, cat	9.1% of each species	Gel	(16)
Multiplex (two-tube)	10	beef, sheep, pork, chicken, turkey; cat, dog, mouse, rat, human	30 pg DNA	Gel	(17)
Multiplex (two-tube)	12	horse, pigeon, camel, rabbit, ostrich, beef; turkey, dog, chicken, duck, cat, goose	0.05-0.1 ng DNA	Gel	(18)
Multiplex (two-tube)	14	cattle, donkey, canidae (dog, fox, raccoon-dog), deer, horse; pig, ovis (sheep, goat), poultry (chicken, duck), cat, mouse	0.02-0.2 ng DNA	Chip	(19)

1. Liu WW, Tao J, Xue M, Ji JG, Zhang YH, Zhang LJ, et al. A multiplex PCR method mediated by universal primers for the identification of eight meat ingredients in food products. *Eur Food Res Technol* (2019) 245:2385-92. doi: 10.1007/s00217-019-03350-9.

2. Cai ZD, Zhou S, Liu QQ, Ma H, Yuan XY, Gao JQ, et al. A Simple and Reliable Single Tube Septuple PCR Assay for Simultaneous Identification of Seven Meat Species. *Foods* (2021) 10. doi: ARTN 108310.3390/foods10051083.

3. Uddin SMK, Hossain MM, Chowdhury ZZ, Johan MR. Detection and discrimination of seven highly consumed meat species simultaneously in food products using heptaplex PCR-RFLP assay. (2021) 100:103938.

4. Wang WJ, Liu JJ, Zhang QD, Zhou X, Liu B. Multiplex PCR assay for identification and quantification of bovine and equine in minced meats using novel specific nuclear DNA sequences. *Food Control* (2019) 105:29-37. doi: 10.1016/j.foodcont.2019.05.016.

5. Hou B, Meng XR, Zhang LY, Guo JY, Li SW, Jin H. Development of a sensitive and specific multiplex PCR method for the simultaneous detection of chicken, duck and goose DNA in meat products. *Meat Sci* (2015) 101:90-4. doi: 10.1016/j.meatsci.2014.11.007.

6. Kim MJ, Yoo I, Yang SM, Suh SM, Kim HY. Development and validation of a multiplex PCR assay for simultaneous detection of chicken, turkey and duck in processed meat products. *Int J Food Sci Tech* (2018) 53:2673-9. doi: 10.1111/ijfs.13876.

7. He H, Hong X, Feng Y, Wang Y, Ying J, Liu Q, et al. Application of quadruple multiplex PCR detection for beef, duck, mutton and pork in mixed meat. (2015) 3:392-8. doi: 10.12691/jfnr-3-6-6.

8. Qin PZ, Qu W, Xu JG, Qiao DQ, Yao L, Xue F, et al. A sensitive multiplex PCR protocol for simultaneous detection of chicken, duck, and pork in beef samples. *J Food Sci Tech Mys* (2019) 56:1266-74. doi: 10.1007/s13197-019-03591-2.

9. Li XN, Guan YF. Specific identification of the adulterated components in beef or mutton meats using multiplex PCR. *J Aoac Int* (2019) 102:1181-5. doi: 10.5740/jaoacint.18-0338.

10. Liu WW, Wang XN, Tao J, Xi BS, Xue M, Sun WP. A Multiplex PCR Assay Mediated by Universal Primers for the Detection of Adulterated Meat in Mutton. *J Food Protect* (2019) 82:325-30. doi: 10.4315/0362-028x.Jfp-18-302.

11. Thanakiatkrai P, Dechnakarin J, Ngasaman R, Kitpipit T. Direct pentaplex PCR assay: An adjunct panel for meat species identification in Asian food products. *Food Chem* (2019) 271:767-72. doi: 10.1016/j.foodchem.2018.07.143.

12. Ali ME, Razzak MA, Abd Hamid SB, Rahman MM, Al Amin M, Abd Rashid NR, et al. Multiplex PCR assay for the detection of five meat species forbidden in Islamic foods. *Food Chem* (2015) 177:214-24. doi: 10.1016/j.foodchem.2014.12.098.

13. Wang WJ, Wang XK, Zhang QD, Liu ZH, Zhou X, Liu B. A multiplex PCR method for detection of five animal species in processed meat products using novel species-specific nuclear DNA sequences. *Eur Food Res Technol* (2020) 246:1351-60. doi: 10.1007/s00217-020-03494-z.

14. Safdar M, Junejo Y. The development of a hexaplex-conventional PCR for identification of six animal and plant species in foodstuffs. *Food Chem* (2016) 192:745-9. doi: 10.1016/j.foodchem.2015.07.082.

15. Matsunaga T, Chikuni K, Tanabe R, Muroya S, Shibata K, Yamada J, et al. A quick and simple method for the identification of meat species and meat products by PCR assay. *Meat Sci* (1999) 51:143-8. doi: 10.1016/S0309-1740(98)00112-0.

16. Xu J, Zhao W, Zhu MR, Wen YJ, Xie T, He XQ, et al. Molecular identification of adulteration in mutton based on mitochondrial 16S rRNA gene. *Mitochondrial DNA A* (2016) 27:628-32. doi: 10.3109/19401736.2014.908377.

17. Prusakova OV, Glukhova XA, Afanas'eva GV, Trizna YA, Nazarova LF, Beletsky IP. A simple and sensitive two-tube multiplex PCR assay for simultaneous detection of ten meat species. *Meat Sci* (2018) 137:34-40. doi: 10.1016/j.meatsci.2017.10.017.

18. Cai ZD, Zhong GW, Liu QQ, Yang XQ, Zhang XX, Zhou S, et al. Molecular Authentication of Twelve Meat Species Through a Promising Two-Tube Hexaplex Polymerase Chain Reaction Technique. *Front Nutr* (2022) 9:813962.

19. Li JC, Li JP, Xu SG, Xiong SY, Yang JN, Chen X, et al. A rapid and reliable multiplex PCR assay for simultaneous detection of fourteen animal species in two tubes. *Food Chem* (2019) 295:395-402. doi: 10.1016/j.foodchem.2019.05.112.
